# P-255. Evaluation of automated disinfectant dispenser systems in 6 hospitals demonstrates a need for improved monitoring to ensure that correct disinfectant concentrations are delivered

**DOI:** 10.1093/ofid/ofae631.459

**Published:** 2025-01-29

**Authors:** Jennifer Cadnum, Claire Kaple, Elie Saade, Amy Ray, Curtis Donskey

**Affiliations:** Northeast Ohio VA Medical Center, Cleveland, Ohio; Case Western Reserve University, Cleveland, Ohio; Case Western Reserve University, Cleveland, Ohio; The Metrohealth System, Cleveland, Ohio; Cleveland VA Hospital, Cleveland, Ohio

## Abstract

**Background:**

Automated dispensers that dilute concentrated disinfectants with water are commonly used in healthcare facilities. However, most facilities do not perform routine monitoring to ensure that the dispensers are functioning correctly and being used as recommended by the manufacturer.

**Table 1.**
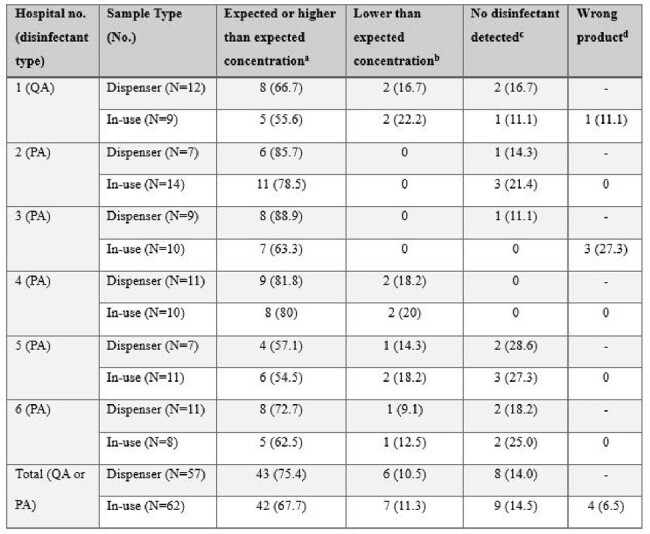
Concentrations of disinfectant measured in samples obtained from automated dispensers and from in-use buckets on environmental services carts in 6 hospitals QA, quaternary ammonium disinfectant; PA, peracetic acid-based disinfectant. a. Expected concentrations, ∼1,300 parts per million (PPM) for PA and ∼800 PPM for QA; higher-than-expected concentration of peracetic acid, >1,800 ppm; b. Lower than expected concentration, 300-900 PPM for PA and 200-400 PPM for QA; cNo disinfectant detected, limit of detection (∼300 PPM for PA and ∼150 PPM for QA); d. Wrong product, the in-use product that was supposed to be PA or QA was a detergent intended for floors.

**Methods:**

In 6 acute care hospitals in Northeast Ohio, we tested disinfectant concentrations and pH of products obtained from automated dispensers and in-use containers from environmental services (EVS) personnel carts. The disinfectants included quaternary ammonium (800 parts per million [ppm]; pH 8) and peracetic acid-based products (∼1300 ppm; pH 3).

**Results:**

All 6 hospitals had 1 or more malfunctioning dispensers (Table 1). Of 57 systems assessed, 14 (25%) dispensed products with lower-than-expected concentrations, including 8 (14%) with no detectable disinfectant (i.e., water). Nine of 62 (15%) in-use products had no detectable disinfectant, and 4 (7%) were the wrong product (i.e., a detergent for floors used for high-touch surfaces). Reasons for dispenser malfunction included incorrect connection of the concentrate container, dysfunction of a low-product indicator, and failure to change the concentrate container when indicated. pH measurements identified disinfectants with lower-than-expected concentrations, and an intervention that included monitoring was effective in ensuring that the dispensers were operating correctly.

**Conclusion:**

Improved monitoring of automated disinfectant dispenser systems in healthcare facilities is needed to ensure patient safety. pH measurements could provide a simple means to ensure that dispensers are functioning correctly.

**Disclosures:**

**Elie Saade, MD, MPH, FIDSA**, Janssen Global Services: Advisor/Consultant|Janssen Global Services: Advisor/Consultant|Janssen Research and Development: Advisor/Consultant|Janssen Research and Development: Advisor/Consultant **Curtis Donskey, MD**, Clorox: Grant/Research Support|Pfizer: Grant/Research Support

